# Competency requirements for patients and therapists in telerehabilitation aftercare: a qualitative study

**DOI:** 10.3389/fresc.2025.1640416

**Published:** 2025-10-23

**Authors:** Anna Lea Stark-Blomeier, Stephan Krayter, Christoph Dockweiler

**Affiliations:** ^1^ Chair of Digital Public Health, Department of Social Sciences, Faculty of Arts and Humanities, University of Siegen, Siegen, Germany; ^2^ Chair of Information Systems, esp. IT for the Aging Society, School of Economic Disciplines, University of Siegen, Siegen, Germany

**Keywords:** rehabilitation, aftercare, telemedicine, telerehabilitation, qualitative research, skills, focus groups

## Abstract

**Introduction:**

Rehabilitation aftercare serves to maintain the success of treatment following medical rehabilitation. Digital services, such as app-based training or therapist-led video calls, are increasingly being used in rehabilitation and aftercare as alternatives that are more flexible in terms of space and time. However, such systems place various demands on users. The study aims to identify the requirements and competencies needed by patients and therapists for the successful use of telerehabilitation aftercare in Germany.

**Materials and methods:**

The study employed an explorative, qualitative approach. Focused interviews were conducted to gather user experiences with telerehabilitation aftercare. Fifteen therapists participated in three focus group interviews, while five patients took part in three individual or two-person interviews. Using an interview guideline, patients and therapists were asked about steps, preparation processes, required competencies and adaptation possibilities in relation to telerehabilitation aftercare. The evaluation was carried out using structuring content analysis according to Kuckartz and Rädiker.

**Results:**

Depending on the program used and the professional background of the therapists, the perceived usage requirements differed, including technical handling, motivation for training and individual therapy adaptations. Both target groups considered application-, process- and impact-related knowledge, technical, social-emotional and cognitive skills, a positive attitude towards technology and technical experience as necessary. Therapists emphasized professional skills as well as experience, and patients sufficient physical skills such as motor skills. The influence of socio-demographic factors on usage was controversially discussed.

**Conclusion:**

Findings suggest to focus not only on technical but also on professional and social competencies in training and further education in order to promote the competent use of telerehabilitation aftercare. If sufficient opportunities are created to get to know and try out such programs, uncertainties could be reduced and positive user experiences can be promoted. Due to the small sample size, the results cannot be generalized without restriction, and further research with a larger and more diverse sample is necessary.

## 1 Introduction

Medical rehabilitation aims at the recovery from physical and mental damages, disorders or diseases (1). It follows a multidisciplinary approach, involving health professionals such as physicians, psychologists, social workers, physiotherapists and occupational therapists to meet the patient's needs (2). Following medical rehabilitation, subsequent aftercare allows patients to continue therapy for example in outpatient clinics and thus ensure that the success of treatment is maintained in everyday life (3, 4). While rehabilitation aftercare is not yet common internationally, in Germany one in four medical rehabilitations is followed by aftercare (5). The digital transformation of healthcare has led to the emergence of innovative digital tools and services aimed at improving medical rehabilitation and rehabilitation aftercare. Telerehabilitation, in this context, encompasses the use of rehabilitative services via information and communication technologies and enables patients to make use of these services from home (6). Current meta-analyses and systematic reviews show that telerehabilitation is equivalent to standard care, for example, regarding functional outcomes for patients with stroke, spinal cord injury, multiple sclerosis, or Parkinson's disease (7, 8). In Germany, telerehabilitation aftercare is part of standard care, with programs falling into three categories: (1) app-based multimodal programs combining at least two therapy areas and offering exercises, lectures, and other therapeutic content across all indication areas; (2) app-based training-therapeutic programs including exercise therapy for postural or musculoskeletal impairments; and (3) psychosomatic video call programs featuring weekly group-based discussions. In addition to the three core programs, there are specialized programs designed for specific patient groups or offered through alternative delivery modes, such as app-based psychosomatic aftercare for depressive patients (9). The use of these services has increased significantly in recent years, with the number of services rising from around 300 in 2019 to almost 19,000 in 2023, 88% of which are multimodal (5). In 2023, the average user was 53 years old and female (56%) (10). However, telerehabilitation aftercare only accounts for around 7% of all aftercare services in Germany (5).

In order to exploit healthcare-related potential in the use of digital health services, specific skills are required for usage (11, 12). For example, people with better health-related or technical skills are more willing to use digital health apps and are more successful in using them (13, 14). The usage requirements and competencies needed for telerehabilitation have not yet been systematically investigated—and so there is a lack of competency profiles or models, such as those already established in the field of telemedicine (15–17). First indications of usage requirements for telerehabilitation can be found in current systematic reviews that describe the available programs and their functions. For example, two reviews (18, 19) on exercise-based telerehabilitation for patients with cardiac or pulmonary diseases show that its usage is associated with a wide range of tasks for users. Thus, it is evident that telerehabilitation programs differ in their training modalities, frequency, duration, and intensity for patients. Depending on the program, patients must use different technologies and software, such as smartphone or tablet apps, websites, or videoconferencing platforms. Patient tasks include motivating oneself for digital training, carrying out the exercises and collecting and forwarding data. Health professionals are responsible for monitoring, providing feedback and consultations, and motivating patients to varying degrees, depending on the program (18, 19). These tasks provide information about possible relevant competencies for telerehabilitation, such as motivational or technical skills, which were also identified as relevant in competency profiles or models for telemedicine professionals (15–17). Competency models have the added value of collecting, systematizing, and structuring necessary competencies. For example, the “Requirements Profile for Non-Medical Assistants in Telemedicine Centers” differentiates between methodical, technical, social and personal competencies (15), and the “Interprofessional Framework for Telebehavioral Health Competencies” structures competencies along different competency areas and levels (16).

An analysis of the requirements and relevant competencies specifically for telerehabilitation is essential because the rehabilitation sector with its involved care actors, processes and needs differs greatly from other care sectors in which telemedicine is used, such as emergency medicine or specialist care, and consequently the digital tools applied and requirements for their usage also vary (20). As shown previously, it must be considered that telerehabilitation is a heterogeneous field of practice that varies in terms of therapy content, digital tools and user groups (e.g., demographic characteristics, differences between the user group of health professionals and patients). Against this background, a broad understanding of the usage requirements is needed that takes into account the different needs and demands of patients and health professionals (21). The study therefore aims to qualitatively assess the requirements perceived by users of telerehabilitation aftercare and to derive necessary competencies. The study is based on two research questions: (1) What are the requirements faced by therapists and patients when using telerehabilitation aftercare?, and (2) What competencies are required for successful usage?

## 2 Materials and methods

### 2.1 Study design

An explorative, qualitative design was used for providing first insights into user experiences in telerehabilitation aftercare. Focused interviews were conducted, which are characterized by the circumstance that all interviewees have experienced a social situation whose subjective perception is to be explored. This method is appropriate to fully understand the specific experiences and personal perceptions of patients and therapists (22, 23). Focused interviews are structured by a guideline, begin with a stimulus (e.g., newspaper article) and can be realized as individual interviews, dyads or focus groups (22, 23). The research project and the methods used in this study were reviewed by the Council of Research Ethics of the University of Siegen (ER_4/2022). This article follows the Standards for Reporting Qualitative Research (24) (see Supplementary File S1).

### 2.2 Recruitment and sampling

When composing groups for focused interviews, it is important to make them homogeneous to ensure an relaxed interview situation that is comforting and facilitates sharing. In this context, homogeneity means that the participants have something in common that relates to the topic of conversation. Thus, in our study we recruited people with experience in telerehabilitation aftercare and conducted separate interviews with the group of patients and the group of therapists (23, 25). Nevertheless, in order to obtain a comprehensive picture of the opinions of this diverse target group, we aimed for contrasting cases in terms of age, gender, indication area and type of therapy (26). Against this background, recruitment was carried out across all indications and programs. Patients were included if they had used a telerehabilitation aftercare program in the last six months. This ensured that they had sufficient memory of their experiences with the program and they were assumed to be in sufficient physical and mental condition to participate in the digital interviews. Furthermore, in Germany, rehabilitation aftercare begins no later than three months after medical rehabilitation, providing a standardized timeframe for patient participation (9). Therapists were included if they were involved in the therapy planning, patient supervision or delivery of telerehabilitation aftercare within the last six months.

The targeted sample size for the focus groups was set at five persons, in line with the recommendations of Krueger and Casey (25). Given the exploratory nature of the study, our goal was not theoretical saturation but rather first insights and a detailed view of needs across a diverse group of telerehabilitation users. To minimize selection bias, the recruitment was conducted via various channels from June to August 2022. For this purpose, (1) all telerehabilitation aftercare facilities and software manufacturers with their own therapists (*n* = 277, see https://www.nachderreha.de, as of July 2022), (2) specific rehabilitation-related websites (*n* = 2) and closed Facebook groups (*n* = 2), as well as (3) all self-help contact centers (*n* = 365, see https://www.nakos.de) in Germany were contacted via E-Mail. These were to forward the information flyer (see Supplementary File S2) directly to the target groups or disseminate it via homepages or newsletters. All interested persons participated in the interviews. Due to low feedback among patients, the focused interviews with them were not realized as focus groups, but in individual or two-person format.

### 2.3 Development of interview guidelines and questionnaires

The semi-structured interview guidelines were developed according to the group-dynamic process of focus groups (27) in five phases (see Table 1). First, one moderator welcomed the participants and introduced him/herself, the objectives, the agenda and the discussion rules. An introduction phase with a warm-up question was followed by a transition question with a discussion stimulus [excerpt from a scientific article (28)]. This was followed by the main questions catalog with content-related questions and a final question. The results of a scoping review (20) were embedded as a deductive element of the guideline development. Competencies were thus defined as knowledge areas, skills, attitudes, experiences and personal characteristics required for successful use (20, 29).

**Table 1:** Excerpt from the interview guideline for patients (translated from German to English).

Phase	Topic	Main question/s
Phase 1: Welcoming	Introduction by moderator	**/**
Phase 2: Warm-Up Question	Introduction of participants	Please introduce yourself briefly. Please name the telerehabilitation aftercare program(s) with which you already have experience and how long you have participated.
Phase 3: Transition Question	Discussion stimulus	As an introduction to the topic, we would like to present two results from a recently published scientific article. The following statements are made there: “*72% of practitioners find that patients are not competent or confident in using telerehabilitation technology. 15% of practitioners think that practitioners themselves are not competent or confident in using it*”*.* How do you feel about these figures and what experiences have you had?
Phase 4: Content Question	Steps	We would now like to understand how the telerehabilitation aftercare program works. Please tell us from start to finish what steps you go through and what the requirements are for you as a patient.
Phase 4: Content Question	Preparation/Experience	We are wondering how you were prepared for the use of telerehabilitation aftercare. Can you tell us more about this? How important do you consider experience in dealing with technology or telerehabilitation aftercare in order to be competent and confident in the use of such programs?
Phase 4: Content Question	Required competencies	What competencies are required for you as a patient to successfully use telerehabilitation aftercare?
Phase 4: Content Question	Relevant attitudes/personality traits	In your opinion, what attitudes towards technology on the part of patients are necessary or conducive to the competent execution of telerehabilitation aftercare? And which personality traits could play a role in this?
Phase 4: Content Question	Technology adaptation	Digital technologies are said to be quite easy to adapt to the individual needs of the target group. To what extent does this apply to telerehabilitation aftercare programs?
Phase 5: Final Question	Ending	Which aspect did you find particularly exciting today or what was particularly important for you?

The guidelines comprised five content-related blocks. First, the steps involved in telerehabilitation aftercare were surveyed. Then the required competencies were assessed, with sub-questions focusing on knowledge and skills. Experience and attitudes/personal characteristics were surveyed as separate blocks due to their scope and specificity. With regard to usage requirements, the last block dealt with adaptation possibilities of the programs. The guidelines were checked for comprehensibility and completeness in a qualitative pretest (30) by a therapist, a therapy manager and a researcher and adapted slightly in terms of language.

In addition, a short questionnaire was created. It assessed socio-demographic factors (age, gender, marital status, educational/vocational qualifications), the used telerehabilitation aftercare program, the indication group which the program is aimed at, the duration of use, and for therapists the type of therapy practiced. Furthermore, we assessed the affinity for technology (TA) according to Franke et al. (31) on a 6-point scale (a higher number indicates a higher TA). Along this scale, individuals can be distinguished according to whether they tend to actively interact with new technical systems or rather avoid intensive interactions. This affects whether users can “figure out” technical systems on their own or need assistance when familiarizing with new technologies (ibid.).

### 2.4 Data collection

The interviews were conducted online via Zoom so that remote participants could take part at a low threshold. Three focused interviews in focus group format with therapists (4–6 participants in each group; *n* = 15 in total) and three focused interviews in individual or two-person format with patients (*n* = 5 in total) took place from September to October 2022. Before the interviews, participants gave their written consent and completed the short questionnaire. The interviews lasted an average of 126 minutes for therapists and 75 minutes for patients. The interviews were audio-recorded. The interview process was the same for both target groups. Participants did not receive vouchers or money for participating.

### 2.5 Data analysis

The audio recordings were transcribed by a transcription agency [simple transcription, slightly smoothed linguistically (32)], pseudonymized, and evaluated using MAXQDA (2024 version; VERBI Software GmbH, Germany) based on the structuring content analysis following Kuckartz and Rädiker (33). The joint analysis by ALS and SK (health scientist and sociologist with experience in moderating/conducting focus groups) made it possible to minimize bias through investigator triangulation (34). The evaluation framework was the deductive-inductive coding system (see Supplementary File S3). Both researcher coded the complete data set independently of each other. Subsequently, all codes were discussed and consented and then transferred to a common coding system. The deductive category development was based on the results of a scoping review (20). The inductive part was based on the interview statements. The short questionnaire was evaluated descriptively using frequency analyses.

## 3 Results

**Figure 1:**
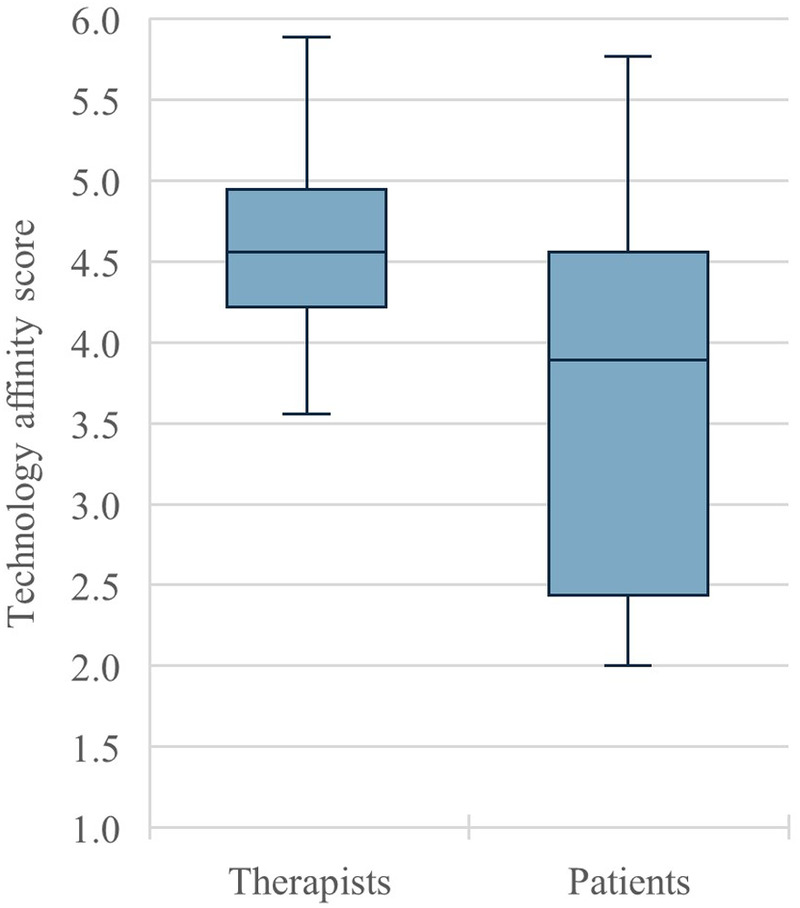
The distribution of technology affinity among therapists (*n* = 15) and patients (*n* = 5).

Fifteen therapists and five patients took part in the interviews (see Table 2). Both target groups had relatively high levels of education (mostly university degrees) and a relatively even gender distribution. The median age of the therapists was 35 years, while the median age of the patients was 54 years. Both samples can be described as rather technology-affine, meaning they tend to actively interact with technical systems and learn or understand technical systems by themselves (see above). Figure 1 shows the different distribution of technology affinity among therapists and patients. It can be seen that therapists had on average a higher affinity for technology, with a median score of 4.6, than patients, with a median score of 3.9. The therapists' affinity for technology shows a smaller range (min = 3.6, max = 5.9) than that of the patients (min = 2.0, max = 5.8), as well as a smaller interquartile range (0.7 for therapists, 2.2 for patients).While therapists mainly used app-based programs for orthopedic aftercare and worked in sports/movement therapy, patients focused on video call programs for psychosomatic aftercare. On average, therapists had 30 months and patients five months of experience with telerehabilitation aftercare.

**Table 2:** Characteristics of the patient and therapist sample.

Variable	Answers	Therapists (*n* = 15)		Patients (*n* = 5)	
		**Median**	**SD**	**Median**	**SD**
Age (years)		35	13.3	54	11.1
Experience (months)		30	16.8	5	1.3
Technology affinity		4.6	0.6	3.9	1.4
		**N**	**%**	**N**	**%**
Gender	Male	7	46.7	2	40.0
	Female	8	53.3	3	60.0
Highest educational/vocational qualification	“Mittlere Reife” (secondary school certificate)	1	6.7	–	–
	“Fachhochschulreife” (specialized A-levels)	1	6.7	–	–
	Vocational training	4	26.7	1	20.0
	Bachelor's or equivalent educational program	3	20.0	1	20.0
	Master’s or equivalent educational program	5	33.3	2	40.0
	Doctoral degree	1	6.7	1	20.0
Type of therapy^a^	Psychotherapy	2	13.3	NA	NA
	Physiotherapy	4	26.7	NA	NA
	Occupational therapy	2	13.3	NA	NA
	Sports/movement therapy	8	53.3	NA	NA
Telerehabilitation aftercare program^a^	App-based multimodal program	13	86.7	1	20.0
	App-based training-therapeutic program	5	33.3	–	–
	Psychosomatic video call program	1	6.7	4	80.0
	App-based psychosomatic program	1	6.7	–	–
Indication group^a^	Mental/psychosomatic disease	4	26.7	4	80.0
	Orthopedics	13	86.7	1	20.0
	Cardiology	8	53.3	–	–
	Neurology	5	33.3	–	–
	Oncology	4	26.7	–	–

^a^ Multiple answers possible; NA, not applicable.

Four therapists with experience in app-based multimodal and partly training-therapeutic programs took part in focus group 1 (FG1), as well as one psychotherapist who had used an app-based psychosomatic program (for the detailed interview composition see Supplementary File S4). The number of contributions to the discussion was fairly evenly distributed. Focus group 2 (FG2) consisted of three physiotherapists, two sports/movement therapists and one psychotherapist. The latter had used a psychosomatic video call program, all others app-based multimodal, partly training-therapeutic programs for mainly orthopedic indications. Three therapists came from one facility and participated from one device. One therapist came from a facility from which another therapist had already participated in FG1. She dominated the conversation (34.9% of all words). In focus group 3 (FG3) all four participants worked in sports/movement therapy and had used app-based multimodal programs. Three therapists each came from a facility from which another therapist had already participated in FG1. One female therapist had the largest share of speech (36.9% of all words). Among patients, two focused interviews (FI1, FI2) were conducted with two psychosomatic patients each using video call programs and the individual focused interview (FI3) was conducted with an orthopedic patient using an app-based multimodal program. In FI1 the contributions were quite evenly distributed, in FI2 the female patient dominated (74.6% of all words).

The content analysis resulted in eight main categories (see Supplementary File S3). The first main category “Usage requirements” describes how steps and tasks differ according to the telerehabilitation aftercare program and which new tasks are associated with this for patients and therapists. The second main category “Preparation for usage” comprises how implementation took place at facility level and how patients as well as therapists were prepared for usage. In the third to seventh main categories, the required competencies, respectively the components “knowledge”, “skills”, “attitudes”, “personal characteristics” and “experiences”, are presented. The eighth main category “Adaptation to needs” describes the extent to which such programs can be individually adapted to the needs of users. This provides information about further usage requirements. The following quotes have been translated from German to English by one researcher (ALS).

### 3.1 Usage requirements

The interviews showed that tasks in telerehabilitation aftercare differ depending on the program. More than two thirds (70.0%) of the interviewees used an app-based program, especially in the orthopedic setting for movement therapy, which included video-based training and educational content for independent usage by patients. In the app, therapists can customize therapy plans via the library and communicate with patients. One therapist supervised an app for depressive patients, in which patients carry out exercises and work on content independently (FG1, T3). Lastly, the psychosomatic video call program was characterized by a group setting in which patients speak with a psychotherapist and do for example mindfulness exercises.

Regarding patient tasks, the programs required independently processing the digital content and carrying out exercises as well as following therapist's instructions. For psychosomatic video call programs, patients noted that they must be able to engage with the group and to actively ask for help with health complaints. For app-based psychosomatic programs, one therapist emphasized creating and following emergency plans (FG1, T3). With app-based programs, patients must be able to request adjustments to treatment plans and to motivate themselves to carry out the exercises. They need to know how to use the program, such as logging onto the platform or navigating the app, and how to deal with technical problems.

The interviewed therapists can be assigned to three job profiles, which are associated with different tasks. There were therapists who work analog in inpatient rehabilitation facilities and are only responsible for the onboarding of aftercare patients. At the end of the patient's stay, they handed over care to teletherapists who provided the telerehabilitation aftercare. “Hybrid” therapists worked both in on-site rehabilitation and in telerehabilitation aftercare. Some therapists (FG1, T3, T10 and T19; FG2, T17) expressed a clear opinion towards their job profile: “*I am a psychotherapist and have expanded my spectrum with digital services. I would not say, I'm a teletherapist*” (FG1, T3). However, the therapists stated that there is still no consensus on the definition or range of tasks in telerehabilitation: “*[…] because nobody really knows what teletherapy is or what it actually involves*” (FG1, T10). In addition, the term teletherapy managers has emerged, who are responsible for patient onboarding or digital therapy planning. According to the interviewees, the tasks of therapists include communication with patients, e.g., for adapting therapy or solving therapy- or health-related problems. Video call programs require the synchronous guidance of the group, app-based programs the development of training plans and monitoring of health-related data. Therapists have to remind patients to carry out the training and motivate them to do so. Finally, therapists were partly responsible for technical support: “*So the therapist was both technical support and an all-rounder*” (FI1, P1).

### 3.2 Preparation for usage

The onboarding of therapists in inpatient rehabilitation facilities took place on-site, or rarely remote via internal training, kick-off events or briefings by the teletherapy clinics, program manufacturers or the rehabilitation facility. In the training courses, the concept of teletherapy and telerehabilitation aftercare, the app/program, its benefits and usage, as well as patient instructions were taught. The introduction of therapists often included independent try-outs and “*learning by doing*” (FG3, T2). This served to familiarize with processes and to anticipate (technical) problems. In one facility, therapists were able to accompany experienced therapists via work shadowing. Some reported that trainees and interns received an introduction (FG2, T5 and T14), while others criticized the lack of integration of telerehabilitation aftercare in vocational training (FG2, T15).

Patient onboarding generally took place during inpatient rehabilitation by therapists working on-site and was then was continued digitally by the same therapists or handed over to a teletherapy clinic. In some cases, there was no on-site introduction (FG1, T10; FG2, T6). While in some facilities only selected patients were informed (FI1, P3; FG2, T16; FG3, T18 and T2), in others everyone received a lecture or was informed via television (FG1, T7 and T19; FI3, P6). Information was also provided via physician's consultation. Partly, patients received individual instructions from therapists or had regular training sessions. It varied whether patients were informed at the beginning or towards the end of the rehabilitation stay. Onboarding by teletherapy clinics usually took place by telephone. For video call programs, it was reported by patients that partly no introduction was given in advance and that the first aftercare session was spent learning how to use it.

Also, the implementation processes in the facilities varied. The interviewees stressed that the manner of implementation determined the success of telerehabilitation aftercare. The therapists explained, that is was not only important to inform therapists, but also other professional groups who come into contact with telerehabilitation aftercare—such as prescribing physicians or social workers. According to the interviewees, telerehabilitation aftercare should be a regular part of the facility's procedures and be “*institutionalized*” (FG1, T3) by being embedded in structures and processes. There were also differences in the extent to which the implementation was structured. While some reported the establishment of a core team, clear distribution of tasks and transparently defined and documented processes (FG3, T2 and T21; FG1, T19), others described a less structured introduction that had to take place quickly due to the coronavirus pandemic (FG1, T7). In general, the onboarding of therapists and patients was considered to be very important, as “*the success [..] of aftercare [..] first depends on how the insured person [..] was introduced to the program*” (FG1, T19).

### 3.3 Required knowledge

The interviewees pointed out that patients and therapists should have application knowledge and thus should know how the program works and how it is set up and used. For patients, this includes knowledge about the download, registration, access and usage. With regard to app-based programs, therapists should be familiar with the content in the library in order to create training plans.

The use of telerehabilitation aftercare is accompanied by new processes that should be known. The interviewees noted that users need knowledge about the processes of telerehabilitation aftercare, as well as the underlying conditions, guidelines and obligations (e.g., regarding cost coverage or data privacy). Patients should understand how care and communication take place. Therapists should also have knowledge of communication processes with patients and participating clinics. Therapists considered it important that responsibilities within the therapist team are clear and that they are informed about innovations regarding therapy, aftercare or the digital programs.

Lastly, according to the interviewees, users should have impact knowledge und thus know about (side) effects, benefits and the effectiveness of telerehabilitation aftercare.

### 3.4 Required skills

The interviewees reported from their experiences that the successful use required basic technical skills in order to handle the programs (e.g., switch on speakers, start video) and solve technical problems. However, patients and therapists stated that the programs were rather simple designed and easy to use: “*[…] I haven't failed with anyone—even though I sometimes really thought ‘okay, it could get bumpy’—because of the technology*” (FG2, T6).

Closely related to technical skills are physical skills, as patients must also be physically capable of operating the technical devices. Barriers to the use of digital movement therapy may exist due to illnesses that impair usage, such as rheumatism or Parkinson's disease, but also other physical limitations such as poor body awareness, limited fine motor skills or poor eyesight.

The interviews indicated that patients and therapists require various social-emotional skills, such as communication skills, for successful usage. For therapists, building a good therapist-patient relationship and digital guidance were discussed: “*[..] in addition to his professional competency, he has to provide guidance in a completely different way. [..] And of course, in the digital setting […] one needs strong communication skills. One must be able to describe things in a vivid manner*” (FG1, T11). During video call sessions, patients and therapists must be able to adhere to conversation rules in order for the therapy to work. This includes, for example, turning off the microphone when not speaking or letting others finish speaking. Furthermore, the therapists mentioned that close teamwork between therapists from different facilities is required to ensure a successful handover and care of patients. With regard to digital therapy, therapists also need empathy: “*We have to be able to put ourselves in the patient's shoes. [..] So, the further away I am from the patient—and we simply are—the better I actually have to be at these things*” (FG1, T10). The need for empathy and teamwork skills was also expressed for patients in video call sessions. In addition, therapists declared that they need to be able to motivate patients to undergo therapy and that patients need to be able to motivate themselves.

Cognitive skills were also discussed as a prerequisite. According to the patients, video call programs require from them a high level of reflection, concentration, the ability to be self-aware and aware of the surroundings, as well as a willingness to adapt to the switch between analog and digital communication. Furthermore, digital training requires patients to have self-management skills. According to the interviewees, therapists should be able to reflect on the benefits and use of telerehabilitation aftercare on an individual basis depending on the patient and anticipate potential health- or usage-related problems. Analytical skills were also seen as important for therapists: “*So, I need to […] have a certain ability to understand connections, and quite quickly. Because otherwise I wouldn't be able to recognize red flags. For example, if I let someone continue training or […] I do not recognize when a person is actually at risk*” (FG1, T10). Especially when starting with telerehabilitation aftercare, users need a good imagination to be able to visualize the usage and benefits. In addition, the introduction requires therapists to be patient with the changes that come with it.

Lastly, the therapists highlighted that telerehabilitation aftercare is a therapeutic service that requires therapeutic-professional skills and should therefore be carried out by trained professionals in accordance with therapeutic and scientific standards. For teletherapists who get to know their patients exclusively digitally, a strong understanding of the patients and their health and home situation is required in order to provide the right care. This includes skills in the recognition, classification and resolution of health complaints. For app-based training therapy, therapists emphasized the skill to convey tactile stimuli or corrections digitally. Some therapists defined a hierarchy of skills and competencies that therapists need: “*Yes, it's quite simple, there is a clear order. First comes personal and professional competency, and then digital competency. And the former is by far more important than the latter. It is also certainly much harder to teach over many, many years*” (FG1, T3).

### 3.5 Relevant attitudes

The interviewees reported from their experience that a positive attitude can promote the successful use of telerehabilitation aftercare. Both target groups therefore require an openness to new things or new technology and an interest in the program. It was stressed that therapists should not only provide telerehabilitation aftercare in order to work remotely, but also because they are convinced of it and enjoy it. It also requires curiosity, courage and a willingness to learn on the part of users. It was summarized that a basic, but not a high affinity for and acceptance of technology is required by patients and therapists.

According to the interviewees, negative attitudes can hinder the successful use of such programs. The interviewees' experiences showed that many health professionals were skeptical or dismissive of telerehabilitation aftercare in the initial phase. This negative attitude could be transferred to the patients and harm successful use, for example by causing uncertainty among patients. There were also patients who generally refused digital therapy or digital tools. According to the interviewees, this skepticism is partly due to prejudices or fears (e.g., of incorrect usage), that could be reduced by trying out the program. The patients' low self-confidence in their own abilities was also rated as an inhibiting factor.

### 3.6 Relevant personal characteristics

The interviewees discussed whether socio-demographic factors influence successful use. Some interviewees were of the opinion that the patients' and therapists' age has no influence (FG1, T7; FG2, T5; FI1, P3), while others attributed greater competencies to younger people (FG2, T6, T16; TG3 T20; FI2, P2). One therapist assumed that patients from rural areas have lower competencies (FG2, T16). A few patients discussed whether a higher level of education (university degree) or a job that involves the use of digital tools could be beneficial in the use of telerehabilitation aftercare by patients (FI1, P1; FI2, P2; FI3, P6). One patient considered a low socio-economic status of patients to be an obstacle if the use of the program is impaired by low-quality hardware due to limited financial resources (FI1, P3).

Other personal characteristics were addressed. The therapists discussed that there are patients with “difficult” personalities for whom the use of telerehabilitation aftercare is challenging—e.g., due to psychological difficulties. Patients pointed out, that some patients are unable to use such programs due to illness.

### 3.7 Relevant experiences

The interviewees explained that experience with technologies in general and health apps can facilitate successful usage by patients. Therapists reported that with growing experience with the program, a routine develops for therapists that facilitates use. Trying programs out and gaining experience also promoted competent use among patients: “*And you could really see it too—in the way they interacted and everything, they became more confident each time. Practice really does make perfect, as the saying goes*” (FI1, P3).

For the provision of telerehabilitation aftercare and especially as a teletherapist, the interviewees pointed out the need for sufficient professional experience in on-site therapy, directly with patients: “*So, I don't think that someone with no professional experience can immediately work as a teletherapist […]. I need to have met people, I need to have seen how things works in my field*” (FG1, T10).

### 3.8 Adaptation to needs

With regard to the programs used, the majority of respondents stated that they were simple and user-friendly and therefore easy and intuitive to use (FG1, T11; FG3, T18; FI1, P3; FI2, P2). Occasionally, the complex or less user-friendly interface was criticized by therapists and patients, as it hinders successful usage (FG2, T5; FG3, T2; FI2, P5).

According to the interviewees, the programs can be individually adapted to the needs of the users to varying degrees. In app-based programs, therapists can create individualized therapy plans and adapt them to the patient (e.g., number of sets, breaks) with the aim of achieving better therapy. One therapist summarized: “*[…] that it's actually more individualized than an [analog] group aftercare at the end*” (FG2, T17). Patients reported on rather limited customization options, e.g., in the speaker view during video calls or the order of exercises in the app, which means that user-friendly handling and usage according to their preferences are only partially possible.

## 4 Discussion

**Figure 2:**
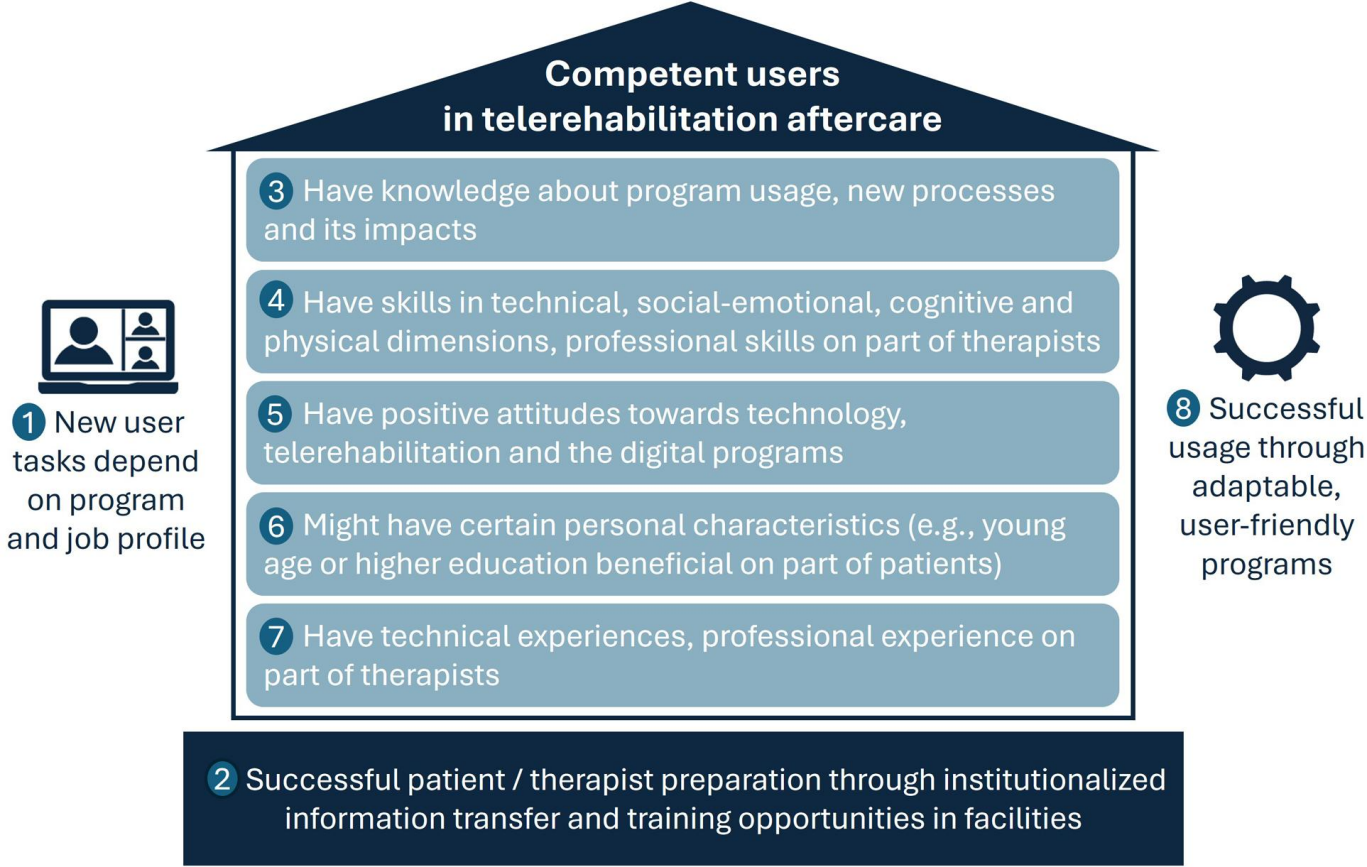
Qualitative interview themes on requirements and competencies in telerehabilitation aftercare.

The content analysis revealed eight thematic categories dealing with requirements and relevant competencies regarding telerehabilitation aftercare (see Figure 2). Based on the sample interviewed, we found that (1) the various tasks and requirements for telerehabilitation aftercare depended on the program used. While for patients using app-based programs, motivation and independent training were mentioned, video call programs required the following of therapist's instructions and active participation. Therapists' tasks varied depending on their job profile. The interviews showed that on-site therapists were responsible for preparing inpatients for aftercare and then handed over digital care to teletherapists. Hybrid therapists provided both analog and digital care. The findings suggest that (2) the foundation for successful telerehabilitation aftercare is a comprehensive preparation of patients and therapists, which is achieved through institutionalization of information transfer and training opportunities in the rehabilitation facilities. The interviewees stated that users need (3) knowledge about the use of telerehabilitation, associated processes, as well as impacts. Personal experiences showed that (4) next to technical skills, users need social-emotional skills such as communication, motivation, empathy and teamwork. Relevant cognitive skills on part of patients were reflection, imagination, concentration, adaptation, self-awareness and environmental awareness, as well as self-management; on part of therapists, reflection, imagination, anticipation, analysis and patience. It stood out that patients had to be physically able to carry out digital therapy, while therapists needed therapeutic-professional skills. (5) Positive attitudes such as openness, interest, conviction, curiosity, courage, willingness to learn, affinity for and acceptance of technology were discussed as beneficial for usage; negative attitudes such as skepticism, rejection, fear and low self-confidence as inhibiting. The respondents reported that (6) technical experience and professional experience among therapists can contribute to successful use. With regard to (7) personal characteristics such as age, there was no consensus among respondents as to whether these could influence the successful use. The above mentioned similarities and differences in the competencies required of patients and therapists are summarized in Table 3. Finally, the interviews showed that (8) users see user-friendly and adaptable digital programs as beneficial for the use of telerehabilitation aftercare.

**Table 3:** Similarities and differences between patients and therapists regarding telerehabilitation competencies.

Component	Patients and therapists	Only patients	Only therapists
Knowledge	• Knowledge on the program and its usage • Knowledge on telerehabilitation and communication processes, underlying conditions, guidelines and obligations • Knowledge on the impact of telerehabilitation	/	• Overview of program content and therapy options • Knowledge on communication processes with clinics, responsibilities and therapy/program innovations
Skills	• Basic technical skills • Social-emotional skills, like communication, teamwork, empathy and motivation • Cognitive skills, like reflection and imagination	• Sufficient physical skills • Concentration, self-management and adaptation skills, self- and environmental awareness	• Analytic skills, anticipation and patience • Therapeutic-professional skills
Attitudes	• Openness to new things/technology and interest in the program • Curiosity, courage and willingness to learn • Basic technology affinity and acceptance	/	• Conviction/belief in the program
Personal characteristics	• Influence of age on usage possible	• Usage difficulties due to psychological/ illness related challenges possible • Influence of place of residence, education, job and socio-economic status on usage possible	/
Experience	• Experience with the program	• Experience with technology and health apps	• Professional experience

The study shows that, on the one hand, telerehabilitation aftercare programs were perceived as easy to use, but on the other hand, there were many requirements for usage. The fact that the use of new digital tools in the healthcare sector goes hand in hand with new job-related requirements for health professionals is also reflected in the development of competency models for telemedicine professionals (15, 16, 35, 36). The competencies relevant for telemedicine are similar to those for telerehabilitation aftercare which we identified. According to Keswani et al., telemedicine core competencies include medical knowledge, communication skills and professionalism (36), and according to Galpin et al. understanding of the use of telehealth, digital communication, professionalism and technical skills (35). What the two frameworks do not address are the experiences and attitudes that according to our study, therapists need in order to practice telerehabilitation. Thus, the interviewed participants pointed out that especially teletherapists working solely digitally must have experience in patient care and therapy on-site to ensure the best possible care and, for example, to recognize health risks at an early stage.

An interesting finding of our study was that therapists considered therapeutic experience and therapeutic competency to be highly relevant and partly regarded these as more important than the necessary digital competencies. At the same time, participants found practical training to be useful in preparation for telerehabilitation aftercare. This is consistent with the findings of the internationally accepted eHealth Capabilities Framework, which describes the knowledge and skills expected of tertiary health graduates in the digital age (37). The education of future health graduates should therefore not be aimed purely at imparting technical skills, but rather at developing adaptable personnel who can initiate change and know how to integrate knowledge and skills into practice. Some of the requirements identified by the therapists in our study are also reflected in the four capability statements from the eHealth Capability Framework. Thus, therapists in telerehabilitation aftercare must also (1) be familiar with digital technologies, systems, and policies (including national guidelines and data security), (2) know how digital programs are integrated into practice and what impact this has on patient care and, for example, patient communication, (3) know how to monitor and care for their patients digitally, which may involve the use of patient data, and (4) actively participate in the implementation of these new programs in their facilities, which involves problem solving, understanding change, and keeping up to date with innovations. Due to the specific characteristics and circumstances of telerehabilitation aftercare in Germany, there are some differences to the eHealth Capabilities Framework. For example, the therapists in our study did not address the various patient-generated data, as big data or machine learning are currently not integrated into the existing aftercare programs. The discussions also did not focus on co-designing or developing innovations, as the subject of the study was programs that are already established in standard care (ibid.). Table 4 summarizes the overlaps, omissions, and new findings of our study compared to the eHealth Capabilities Framework.

**Table 4:** Comparison of the study results with the eHealth capabilities framework by Brunner et al. (37).

Capability Statement	Overlaps with our study	Omissions in our study	Novel insights from our study
1. Digital Technologies, Systems, and Policies: Understand the purpose and function of digital health technologies and systems implemented at local, state or national level, including consideration of legal, policy, and ethical implications.	Our study suggests, that telerehabilitation therapists must be familiar with digital technologies, systems, and policies (including national guidelines and data security) and be able to use these and to anticipate usage problems.	Key components of digital health systems, like electronic health records, as well as ethical implications were not discussed in our study.	The telerehabilitation therapists in our study found the digital programs easy to use and partly rated technical skills as less important.
2. Clinical Practice and Applications: Integrate digital health into clinical practice to deliver safe and quality care, including provision of best practice models of care.	According to our study, therapists must know how telerehabilitation programs are integrated into practice and what impact this has on patient care (e.g., patient-orientation), patient communication, the professional role and patient engagement (e.g., the increased need for self-motivation).	Accessing and aggregating digital data from various sources, as well as evaluating it, were not discussed in our study.	According to our findings, the successful integration of telerehabilitation in practice depends not only on knowledge of the impacts of the digital program, but also on the attitude of the therapists. They should be open and curious about new things, interested in the program, convinced of its benefits, courageous in trying it out, and willing to learn.
3. Data Analysis and Knowledge Creation: Use data and data analysis to inform, deliver, and improve health and health care practice at individual, team, and systems levels.	We found that telerehabilitation therapists must know how to monitor and care for their patients digitally, which may involve the use of patient data.	The range, purpose and potential of various digital health data and data sources (e.g., big data or machine learning), as well as their statistical analysis were not discussed in our study.	Our interviews suggest that, for the digital monitoring of patients and analysis of patient data, it is essential that telerehabilitation therapists have analytical skills, therapeutic expertise and experience in treating patients in order to quickly identify and resolve health risks on the part of patients undergoing digital training.
4. System and Technology Implementation: Participate in digital health implementation, evaluation, and co-design processes to drive improvement and stimulate change.	The therapists of our study stated that they actively participated in the implementation of the telerehabilitation programs in their facilities, which required problem solving, understanding change, and keeping up to date with innovations.	The co-designing or developing of healthcare innovations was not discussed in our study.	Our study shows that it is not only the skills or attitudes of therapists that are necessary for the successful implementation of telerehabilitation, but that all stakeholders involved in telerehabilitation in the facilities must be informed and that the implementation processes must be well-structured.

In general, the use of competency models goes back to the development of competency-based medical education (38). Accordingly, the focus of teaching is no longer on facts only (39), but on the achievement of individual competencies, the performance of learners and practice-oriented learning (38). The present study provides preliminary findings for the development of competency-based curricula in the field of telerehabilitation. An important finding is that the tasks and requirements of the therapists in our study varied depending on the program used and area of application. For example, the use of app-based programs in training therapy—such as physiotherapy—required different competencies than conducting synchronous video call sessions—for example in psychotherapy. At the same time, the study shows that, in addition to imparting theoretical knowledge, other types of education were needed by the interviewees, such as practical try-outs of digital programs, to anticipate usage problems or overcome fears of use. A recent study from Colombia supports these findings and shows that an educational intervention on telerehabilitation, specifically tailored to the structures and challenges in the field of physiotherapy and combining various educational strategies in a blended format with theoretical and practical teaching methods, is effective in increasing knowledge about telerehabilitation among physiotherapists (40). In the future, the user requirements for telerehabilitation aftercare, which were explored in our study, should be expanded and validated in larger-scale quantitative surveys with a larger and more diverse group of participants. As part of a multi-step modelling process, competency models should be developed that systematize the required competencies and visualize relations (29). These should be incorporated into the vocational and continuing training of therapists.

The interviews show that there was no consensus among the interviewees on the definition and design of jobs in telerehabilitation. A transdisciplinary exchange between health professionals, manufacturers, professional associations and other political players is required in order to sharpen the job concepts and range of tasks. It is important that the requirements for digital services in rehabilitation are also embedded in national guidelines and concepts. In Germany, for example, this would be the “Digital rehabilitation aftercare” concept of the German Pension Insurance (9). In addition, professional associations and medical boards are called upon to position themselves with regard to the changing therapeutic job requirements and to help shape new job activities (41).

As described, the practical implementation of preparing for telerehabilitation aftercare in the facilities varied. Participants reported on the lack of standardized and comprehensive information for patients about digital services and rehabilitation aftercare in general. Partly, information deficits are already due to a lack of knowledge on the part of the health professionals involved in the rehabilitation process (including physicians in practices and clinic staff) (42), which must be eliminated in the future. If training offers are not implemented comprehensively, training needs are not met. This highlights the importance of standardizing training processes. However, the personal preconditions and previous experiences of the users in our study were heterogeneous which makes individual onboarding necessary. This could take the form of facilities and health professionals using guidelines and checklists as a basis for their information dissemination and training in telerehabilitation aftercare in order to standardize onboarding processes. At the same time, individual appointments with patients who have opted for or registered for telerehabilitation could be used to ask about their previous experiences and other personal characteristics (e.g., regarding the use of digital tools or motivational skills) in order to subsequently develop and implement a tailored training plan.

The results suggest that initial skepticism and a lack of self-confidence might inhibit successful usage of telerehabilitation, but that these could be reduced by trying the programs out. In future, it will be important to create points of contact with telerehabilitation so that users can familiarize themselves with the program. By introducing it at an early stage during the rehabilitation stay, patients can be given sufficient time to try it out and decide whether or not to use it. The creation of spaces for testing and experiencing has also proven effective in other care contexts, e.g., assistive technologies in the nursing sector (43), in order to support decision-making and adoption processes in the use of new digital technologies.

### 4.1 Strengths and limitations

This study was the first to qualitatively survey the user requirements and competencies needed for telerehabilitation aftercare. The method of focused interviews was suitable for exploring user experiences. Due to a lack of feedback, the focused interviews with patients could not be carried out as focus groups, which is why they were conducted in a one-to-one or two-person format. Although focus groups go back to the data collection method of focused interviews and can be understood as a form of this, they are different data collection methods that differ in terms of composition and group dynamics (23) and therefore significantly influence the nature and richness of data. Accordingly, in individual or two-person interviews, group interaction is either impossible or limited, which means that potential synergy effects in the generation of new ideas and results between participants cannot occur, as these arise from the interplay of different opinions and perspectives (44). This should be taken into account when interpreting the results from the patient interviews.

Although an explorative survey was carried out, which did not seek theoretical saturation, central themes that reflect the user experiences in the context of telerehabilitation were found repeatedly. Nevertheless, the small number of patients is a limitation and the results obtained cannot be generalized accordingly and refer to the sample and context described. The use of incentives or the analog recruitment and conducting of interviews, e.g., in the facilities of the self-help centers, might have achieved a higher response rate. Further surveys with larger and more diverse patient samples are needed to validate the results.

It should be emphasized that the samples do not represent the total population and results are therefore not generalizable. Although the participants mostly used multimodal app-based programs, as the total population does, and the gender ratio and average age of the patients are also comparable (5, 10), it was not possible to recruit older patients (over 63 years), for example, and some programs and indications (e.g., training therapy for orthopedic patients) could only be covered by one person's experience reports. Also, the reported differences between user groups and programs are exploratory findings that do not show causality and would need to be further investigated in quantitative analyses.

Lastly, there may be a selection bias, as patients could not be contacted directly due to a lack of contact details and voluntary participation may have led to the participation of people who are particularly interested in research or particularly technology-affine. Since there are no studies on the technology affinity of users of telerehabilitation aftercare in Germany, no statement can be made about the generalizability of the results. However, it can be assumed that users of telerehabilitation aftercare tend to be more technology-affine, as they have already opted for digital therapy instead of analog therapy in the past.

## 5 Conclusions

The competencies required for telerehabilitation aftercare were not exclusively in the technical area in the sample, but were divided into social-emotional, professional and personal competencies, which also include basic attitudes and experiences. According to the interviewees, training offers and processes were not standardized and in some cases insufficiently institutionalized, which meant that the required competencies were not strengthened as needed. The results suggest that onboarding processes and training offers should take into account the different requirements for telerehabilitation on the part of patients and therapists in the future and be adapted to the needs of the respective user group. This could promote the successful implementation of telerehabilitation aftercare.

## Data Availability

The raw data supporting the conclusions of this article will be made available by the authors, without undue reservation.
