# Supplementary material for: Competency requirements for patients and therapists in telerehabilitation aftercare: a qualitative study
Source: Front Rehabil Sci. 2025 Oct 23;6:1640416. doi: 10.3389/fresc.2025.1640416 (PMC12588949; doi:10.3389/fresc.2025.1640416)
Supplement: Supplementary file 1 [file Datasheet1.pdf]

Supplementary File S3. Coding system

Table S1. Coding system with codes, definitions and quotes

**Main category**	**Subcategory**	**Definition**	**Anchor example / quote**
Usage requirements	Differences according to programs	Interviewees report that the various digital programs are associated with different tasks.	„[...] a mindfulness exercise at the beginning [...] And yes, then the therapist [...] considered a topic to be acute or so relevant that we made it a topic of conversation [...].“ (FI2, P2)
			„[The app] is divided into two parts, so one half is exercises and the other [...] lectures.“ (FI3, P6)
	Patient tasks	Interviewees report that telerehabilitation aftercare brings new tasks for patients.	„But I think when I look at my [video-call] group, even those who are fitter, yes, they have a bad mic and suddenly it doesn't work either. And then they just go back in and then it works.“ (FI1, P1)
	Therapist tasks/ job profiles	Interviewees report that telerehabilitation aftercare brings new tasks/professions for therapists.	„That's also my day-to-day work [as a tele-therapist]. So that means that I work on training plans, have feedback sessions and answer questions in the therapist chat.“ (FG1, T10)
Preparation for usage	Onboarding of patients	Interviewees report how patients were prepared for the use.	„[...] I had an initial consultation with the therapist and in the initial consultation she naturally asked me about the technical modules or whether I had any questions.“ (FI1, P1)
	Onboarding of therapists	Interviewees report how therapists were prepared for the use.	„We [therapists] actually tried out a lot at the beginning, yes. [...] So when we then drew up the plans, or the set-up of the patients and so on [...].“ (FG2, T15)
			„[...] with [...] another colleague, who is responsible for telecare, simply had an introduction to how the program works, how we proceed, what we do there“ (FG3, T18)
	Implementatio n within facilities	Interviewees reported on how the implementation of the programs took place at the facility level.	„You should also get the whole team involved. [...] Which is why we said, okay, physicians also need to get in touch with the system as a matter of principle“ (FG1, T19)
			„You have to institutionalize things. [...] It has to be part of the clinic's procedures“ (FG1, T3)
Required knowledge	Application knowledge	Interviewees report that knowledge of how to use the programs is required.	„[...] the first thing that the patient then knows [...] how this Tele-IRENA [multimodal program] works. [...] i.e. how the app works“ (FG1, T10)
	Process knowledge	Interviewees report that knowledge about the processes of the programs is required.	„And otherwise to adjust [as a therapist] to how some processes work. So, clearly this issue of who is responsible for what, who knows about what.“ (FG2, T7)

	Impact knowledge	Interviewees report that knowledge of the effects/benefits of the programs is needed.	„So that you [as a therapist] understand the advantages that this teletraining can have. [...] what can such a form of therapy do? What can it trigger?“ (FG2, T17)
Required skills	Technical skills	Interviewees report on how technical skills promote successful use.	“So [as a patient] you have to be able to start a video on your cell phone.” (FI3, P6)
			"[...] that as a therapist, if something doesn't work, you don't immediately say, yes, it doesn't matter now. [...] But [...] have understanding, okay, we'll update the page now" (FG2, T14)
	Social-emotional skills	Interviewees report how social-emotional skills promote successful use.	“[...] Motivational work is of course also a big issue because those [patients] are of course much more encouraged to keep track of their appointments themselves [...]” (FG3, T21)
			"And if you're now in a digital setting, of course [...] you have to be a strong communicator. You have to have the ability to describe things visually [...]." (FG1, T11)
	Therapeutic-professional skills	Interviewees report how therapeutic-professional skills promote successful use.	" Say, there is a basic guideline for rehabilitation aftercare. It states the requirements for aftercare therapists. [...] These are the professional requirements, and there's nothing to shake about [...]. Nothing for newcomers to the profession, definitely not." (FG1, T3)
	Cognitive skills	Interviewees report on how cognitive skills promote successful use.	"You also need a certain ability to reflect in order to be able to accept criticism. So not to devalue straight away, but to listen to what others say [...]." (FI1 , P2)
			“So it requires an incredible amount of attention, concentration [...].” (FI1 , P2)
	Physical skills	Interviewees report how physical skills promote successful use.	"[...] there are diseases if someone had something on their hands. [...] Because you have to have fine motor skills to operate this thing." (FI3, P6)
Relevant attitudes	Positive attitudes	Interviewees report an attitude that promotes successful use.	“Yes, so it clearly requires a certain openness [on the part of therapists], openness to new things, the courage to leave gaps, because learning by doing is also important.” (FG3, T2)
			“And personal competence [for therapists] includes many things, [...] ultimately also a certain amount of motivation and enjoyment in what they do.” (FG1, T3)
	Negative attitudes	Interviewees report an attitude that makes successful use more difficult.	“And you also noticed that for a lot of [patients] this fear of this technology inhibited them from even taking sufficient notice of this therapy.” (FI1, P3)
			“[...] almost all [patients] said at the beginning that I didn't think I could do it.” (FG2, T6)
Relevant personality traits	Socio-demographic factors	Interviewees report how socio-demographic aspects promote or inhibit successful use.	“It may be difficult for older people who are not at all [...] media-savvy.” (FI2, P2)
			"[...] the farmer who comes from the countryside, who has no internet [...]. And doesn't have a smartphone either. He might not be able to cope with it in the same way as the patient from the city." (FG2, T16)

	Personal characteristics	Interviewees report how personal characteristics promote or hinder successful use.	"Or just really difficult personalities who are fundamentally unhappy with everything. And then everything that's new is difficult at first." (FG2, T17)
Relevant experience	Experience with technology	Interviewees report how experience with technology promotes successful use.	“But I think it's very, very important that the better and the more often you do it [telerehabilitation aftercare], the more routine you get [as a therapist].” (FG2, T5)
	Professional experience	Interviewees report how professional experience promotes successful use.	"Well, I need a certain amount of professional experience [as a therapist]. I need to have met people, I need to have seen how it works in my field." (FG1, T10)
Adaptation to needs	User-friendliness	Interviewees report how user-friendly/ simple the digital programs are.	"And many [patients] actually find that dealing with it, at least with our [...] program, is actually relatively easy. And they manage it really well." (FG2, T14)
			"[...] it was self-explanatory for me. If it says here, camera, join or a link to, that I click on it and that I then release the microphone and [...] look into the camera." (FI1, P1)
	Individual adaptability	Interviewees report how the digital programs can be adapted to user needs.	“[...] the whole thing is then [...] accompanied by chat, email or even a phone call in between, so that if adjustments are necessary, they can be made accordingly.” (FG3, T21)
