# Supplementary material for: Competency requirements for patients and therapists in telerehabilitation aftercare: a qualitative study
Source: Front Rehabil Sci. 2025 Oct 23;6:1640416. doi: 10.3389/fresc.2025.1640416 (PMC12588949; doi:10.3389/fresc.2025.1640416)
Supplement: Supplementary file 2 [file Datasheet2.pdf]

Supplementary File S4. Interview composition

Table S2. Composition of interviews

No.	Alias	Gender	Age	Program	Indication group	Therapy type
FG1	T3	m	66	Psychosomatic app-based aftercare	Mental/psychosomatic disease	Psychotherapy
	T7	w	30	Multimodal and training-therapeutic app-based aftercare	Orthopedics, Cardiology	Sports/movement therapy
	T10	w	41	Multimodal app-based aftercare	Orthopedics, Cardiology, Neurology	Occupational therapy
	T11	m	46	Multimodal app-based aftercare	Mental/psychosomatic disease, Orthopedics, Cardiology	Sports/movement therapy
	T19	m	35	Multimodal and training-therapeutic app-based aftercare	Orthopedics, Cardiology, Neurology, Oncology	Physiotherapy
FG2	T5	w	53	Multimodal and training-therapeutic app-based aftercare	Orthopedics, Neurology	Physiotherapy
	T6	w	64	Psychosomatic video-call aftercare	Mental/psychosomatic disease	Psychotherapy
	T14	w	24	Multimodal and training-therapeutic app-based aftercare	Orthopedics	Physiotherapy
	T15	m	35	Multimodal app-based aftercare	Orthopedics	Sports/movement therapy
	T16	m	23	Multimodal app-based aftercare	Orthopedics	Physiotherapy
	T17	w	38	Multimodal app-based aftercare	Orthopedics, Cardiology, Neurology, Oncology	Sports/movement therapy
FG3	T2	w	29	Multimodal and training-therapeutic app-based aftercare	Orthopedics, Cardiology	Sports/movement therapy
	T18	m	29	Multimodal app-based aftercare	Orthopedics	Sports/movement therapy
	T20	m	25	Multimodal app-based aftercare	Mental/psychosomatic disease, Orthopedics, Cardiology	Occupational therapy, Sports/movement therapy
	T21	w	47	Multimodal app-based aftercare	Orthopedics, Cardiology, Neurology, Oncology	Sports/movement therapy
FI1	P1	w	54	Psychosomatic video-call aftercare	Mental/psychosomatic disease	NA
	P3	w	37	Psychosomatic video-call aftercare	Mental/psychosomatic disease	NA
FI2	P2	w	54	Psychosomatic video-call aftercare	Mental/psychosomatic disease	NA
	P5	m	34	Psychosomatic video-call aftercare	Mental/psychosomatic disease	NA
FI3	P6	m	63	Multimodal app-based aftercare	Orthopedics	NA

NA = not applicable
