# Supplementary figures and images for: Competency requirements for patients and therapists in telerehabilitation aftercare: a qualitative study
Source: Front Rehabil Sci. 2025 Oct 23;6:1640416. doi: 10.3389/fresc.2025.1640416 (PMC12588949; doi:10.3389/fresc.2025.1640416)

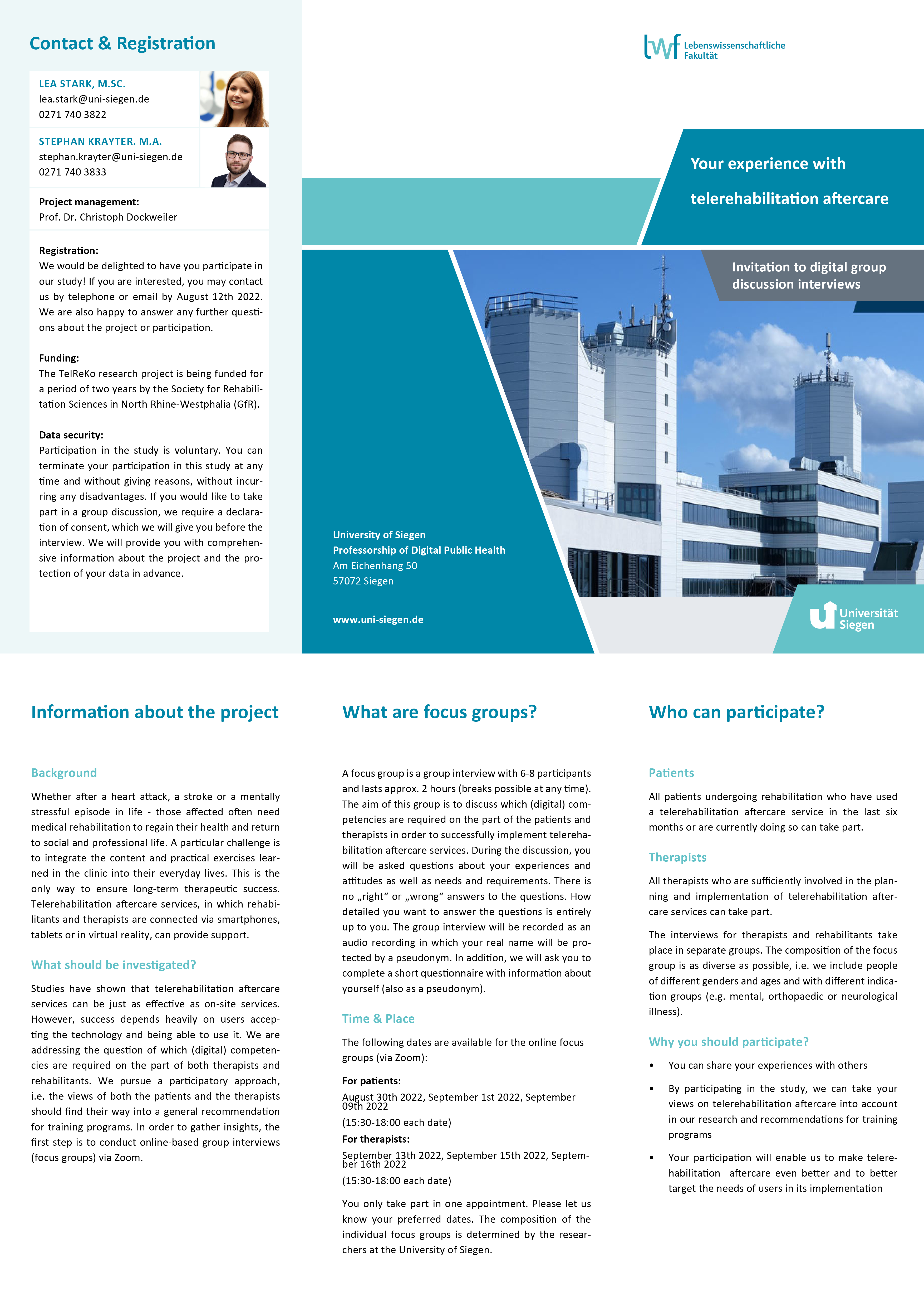

Supplement: Supplementary file 4 [file Image1.png]
